# Effects of Mitragynine and a Crude Alkaloid Extract Derived from *Mitragyna speciosa* Korth. on Permethrin Elimination in Rats

**DOI:** 10.3390/pharmaceutics7020010

**Published:** 2015-03-27

**Authors:** Kachamas Srichana, Benjamas Janchawee, Sathaporn Prutipanlai, Pritsana Raungrut, Niwat Keawpradub

**Affiliations:** 1 Department of Pharmacology, Faculty of Science, Prince of Songkla University, Songkhla 90112, Thailand; E-Mails: hungayonko_o@hotmail.com (K.S.); sataporn.p@psu.ac.th (S.P.); 2 Natural Product Research Center, Faculty of Science, Prince of Songkla University, Songkhla 90112, Thailand; 3 Department of Biomedical Sciences, Faculty of Medicine, Prince of Songkla University, Songkhla 90112, Thailand; E-Mail: rpritsan@medicine.psu.ac.th; 4 Department of Pharmacognosy and Pharmaceutical Botany, Faculty of Pharmaceutical Sciences, Prince of Songkla University, Songkhla 90112, Thailand; E-Mail: niwat.k@psu.ac.th

**Keywords:** kratom, pyrethroids, toxicokinetic interaction, metabolic ratio

## Abstract

Detoxification and elimination of permethrin (PM) are mediated by hydrolysis via carboxylesterase (CES). *Mitragyna speciosa* (kratom) contains mitragynine (MG) and other bioactive alkaloids. Since PM and MG have the same catalytic site and *M. speciosa* is usually abused by adding other ingredients such as pyrethroid insecticides, the effects of MG and an alkaloid extract (AE) on the elimination of PM were investigated in rats. Rats were subjected to single and multiple pretreatment with MG and AE prior to receiving a single oral dose (460 mg/kg) of PM. Plasma concentrations of trans-PM and its metabolite phenoxybenzylalcohol (PBAlc) were measured. The elimination rate constant (k_el_) and the elimination half-life (*t*_1/2 el_) of PM were determined, as well as the metabolic ratio (PMR).A single and multiple oral pretreatment with MG and AE altered the plasma concentration-time courses of both trans-PM and PBAlc during 8–22 h, decreased the PMRs, delayed elimination of PM, but enhanced elimination of PBAlc. Results indicated that PM–MG or AE toxicokinetic interactions might have resulted from the MG and AE interfering with PM hydrolysis. The results obtained in rats suggest that in humans using kratom cocktails containing PM, there might be an increased risk of PM toxicity due to inhibition of PM metabolism and elimination.

## 1. Introduction

**Figure 1:**
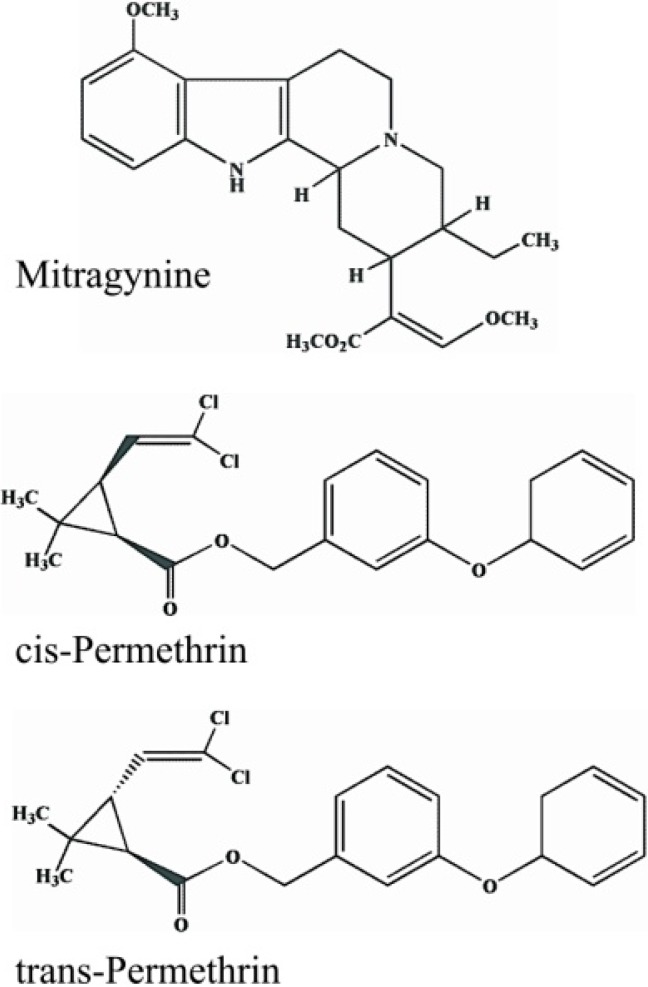
Chemical structures of mitragynine and permethrin (*cis*- and *trans*-isomers).

*Mitragyna speciosa* Korth. (Rubiaceae), known as “kratom” in Thai, is indigenous to Thailand and other countries in Southeast Asia [1]. In the past, leaves of *M. speciosa* were chewed as an opium substitute before it was banned [2]. Thai villagers in the South have used it for a long time as a traditional medicine to relieve tiredness and muscle fatigue, and to treat some common illnesses such as diarrhea, coughing, muscle pain, diabetes, and hypertension [3,4]. It is representative of a particular social and local culture in southern Thailand. However, it has negative health impacts such as withdrawal symptoms due to addiction [4]. Health benefits of *M. speciosa* are due to the stimulatory and opioid-like effects of its bioactive constituents, such as a major alkaloid mitragynine (MG; Figure 1) and a minor component 7-hydroxymitragynine [5,6]. Several studies have revealed a number of pharmacological effects of *M. speciosa*, such as antinociception, antidiarrhea, antiinflammation, inhibition of gastric acid secretion, and stimulation of glucose transport in muscle cells [5,6,7,8,9,10]. In addition, it has been reported that it is used to treat opioid withdrawal [11].

Misuse of *M. speciosa* is especially found in Southern Thailand among adolescents. Users consume it in a form of cocktail, for example the“4 × 100” formulae containing boiled leaf extract, cola beverages, and codeine- or diphenhydramine-containing cough syrup as the basic ingredients. Other components are mosquito coils, anxiolytic drugs, abuse drugs such as methadone and methamphetamine, herbicide, or the powder peeled from the inside of fluorescent light bulbs. A toxicological analysis of the blood and urine to determine the cause of death of one young man revealed the presence of several substances found in “4 × 100” such as MG, alprazolam, nortriptyline, tramadol, and methamphetamine. Such a combination of multiple drugs can result in additive or synergistic adverse effects, especially depression of the central nervous system and the respiratory system, and that was probably the cause of his death [12].

**Figure 2:**
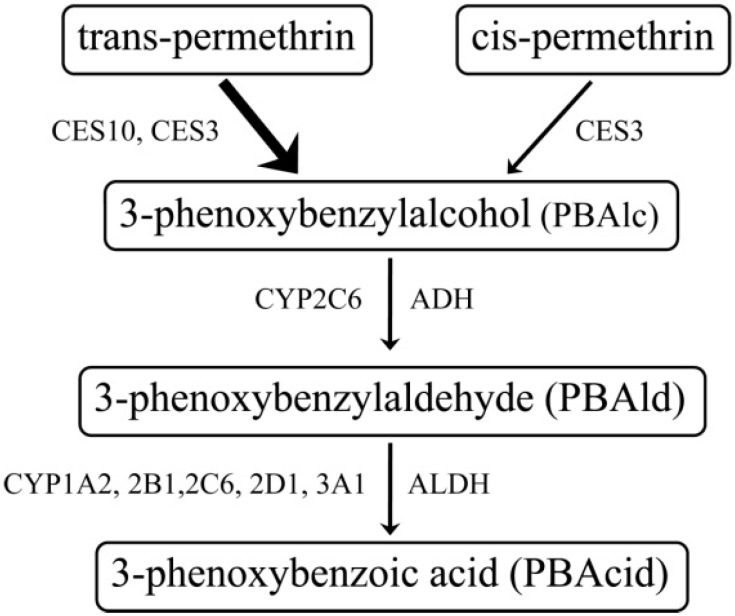
Scheme representing the metabolic pathway of permethrin in rat liver [16].

A mosquito coil usually contains synthetic pyrethroid insecticides, substances structurally modified from pyrethrum extract. Common features of all pyrethroids include the presence of an acid moiety (central ester bond) and an alcohol moiety, and they are usually present as a stereoisomer. Pyrethroids are divided into two subclasses: type I (e.g., allethrin, permethrin (PM; Figure 1) and type II (e.g., deltamethrin, cypermethrin), based on the absence or presence, respectively, of a cyano group at the α-carbon of the alcohol moiety [13]. The toxic effects of pyrethroids on insects and mammals are produced by disrupting the function of voltage-sensitive sodium channels in nerve cell membranes.In rodents, type I-pyrethroids produce the T (tremor) syndrome, while type II-congeners generate CS (choreoathetosis with salivation) syndrome [14]. Detoxification of pyrethroids such as permethrin (Figure 2) in mammals depends on its rapid hydrolysis by carboxylesterase (CES) to the constituent alcohol, which is further oxidized to an aldehyde, then to a carboxylic acid in the liver, prior to excretion into the urine and feces [14,15].

Investigation of the metabolites of MG in rats by liquid chromatographic mass spectrometry has indicated that MG was metabolized via hydrolysis of the methylester [17]. It may be hypothesized that MG affects the hydrolytic inactivation of pyrethroids, causing a delay in their elimination. That may increase the risk of toxicity of a synthetic pyrethroid in kratom cocktail users. A common synthetic pyrethroid used in mosquito coils is allethrin, yet PM is used since it is more photostable with comparative insecticidal activity. Since PM detoxification via hydrolysis occurs both in humans and rats, the present study was therefore meant to investigate any effects from MG and alkaloid extract (AE) derived from leaves of *M. speciosa* on the elimination of PM in rats.

## 2. Experimental Section

### 2.1. Chemicals and Reagents

Standard PM (purity 98%; a mixture of *cis*- and *trans*-isomers; 26.4:71.7; PESTANAL^®^) and phenoxybenzyl alcohol (PBAlc; purity 98%) were from Sigma–Aldrich (Steinheim, Germany) and Aldrich (St. Louis, MO, USA), respectively. Methanol and acetonitrile (HPLC grade), and perchloric acid (AR grade) were from Mallinckrodt Baker Inc. (Phillipsburg, NJ, USA). Chloroform (AR grade) was from VWR International (Fontenay-sous-Bois, France). Acetic acid (AR grade) was from Merck (Darmstadt, Germany). Heparin (NUPARIN^®^) was from Troikaa Pharmaceuticals (Gujarat, India).

### 2.2. Plant Alkaloids

Crude AE and MG were obtained from previous studies [18,19]. Leaves of *M. speciosa* were dried and extracted with methanol and chloroform with a pH modification to yield crude AE, which was further separated by using normal phase column chromatography. The pure compounds were identified by using MS (Thermofinnigan MAT 95 XL mass spectrometer: EIMS with direct insert probe; Thermo Finnigan MAT GmbH, Bremen, Germany), and ^1^H-NMR and ^13^C-NMR (Varian Unity Inova 500 NMR spectrometer, McKinley Scientific, Sparta, NJ, USA) spectra. Procedures for the extraction and isolation of crude alkaloids and MG have been described in detail elsewhere [18,20,21].

### 2.3. Animals

The experimental protocol was approved by the Animal Ethic Committee for Experimental Animals, Prince of Songkla University (Ref. 13/2012, approved 29 March 2012). Animal handling was in accordance with the guidelines of the National Research Council of Thailand based on the International Guiding Principles for Biomedical Research Involving Animals [22]. The experimental work also followed the ARRIVE guidelines [23]. Adult male Wistar rats (Mlac:WR; 200–220g) were obtained from the Southern Laboratory Animals Facility, Prince of Songkla University, Songkhla, Thailand. They were housed in a controlled environment (*ca*. 23 °C, 12 h dark/light cycles) with food and water *ad libitum*. They were fasted overnight with free access to water prior to treatment.

### 2.4. Experimental Design

Four groups of animals (*n*=6 each) were assigned different pretreatments; gr. I, MG 40 mg/kg, *per os* (p.o.), once; gr. II, AE 100 mg/kg, p.o. once; gr. III, MG 40 mg/kg, p.o., 4 days; gr. IV, AE 100 mg/kg, p.o., 4 days. MG and AE were prepared by dissolving in 20% tween 20. The animals of each group were subjected to two phases of the experiment. In phase I, they were given a single oral dose of 460 mg/kg of PM prepared in corn oil. In phase II, the same animal was pretreated with either MG, or AE, 2 h before receiving the same dose of PM. The wash out period was 5 days.

Blood samples were periodically collected by tail clipping without anesthesia at 0, 8, 14, 18 and 22 h after PM administration in phase I and phase II. One-half a milliliter of blood sample was placed in an ice-chilled microcentrifuge tube containing 50 μL of heparin (60 unit/mL) and 2.5 μL of perchloric acid. After thoroughly mixing, it was centrifuged at 1600×*g* at 4°C for 15 min. Plasma was separated and kept at −20°C prior to analysis.

### 2.5. Determination of Permethrin (PM) and Phenoxybenzylalcohol (PBAlc) in Plasma

#### 2.5.1. Preparation of Standard Solutions

An individual stock solution of 10 mg/mL of PM and PBAlc was prepared by dissolving with acetonitrile. Working standard solutions of PM and PBAlc were prepared as a mixture by diluting the stock solution with acetonitrile to different concentrations that ranged from 0.5 to 1024 µg/mL. Standard solutions for calibration at concentrations of 0.1, 0.2, 0.4, 0.8, 1.6, 3.2, 6.4, 51.2, 102.4, and 204.8 µg/mL were prepared by adding the appropriate concentrations of the working standard solutions to plasma blank samples (final volume 200 µL).

#### 2.5.2. Sample Extraction

Plasma samples were extracted using the solid phase extraction (SPE) method with some modifications [24]. Samples (200 µL) were thoroughly mixed by vortex with 60 µL of 1 N acetic acid. The VertiPak™ C_18_-Tubes (200 mg/3mL, Vertical Chromatography, Nonthaburi, Thailand) cartridges were preconditioned with3 mL of chloroform, methanol, methanol–water (50%, *v*/*v*), and water. The cartridges were washed with 3 mL of methanol–water (10%, *v*/*v*), and eluted with 3 mL of chloroform. The eluate was evaporated to dryness with a stream of nitrogen gas at room temperature. The residue was reconstituted with 200 µL of acetonitrile and a 20 µL aliquot was injected into the HPLC system.

#### 2.5.3. Chromatographic Instruments and Condition

High performance liquid chromatography (HPLC) was performed using a Waters 2695 Separation Module connected with a Waters 5487 Dual λ Absorbance detector (Milford, MA, USA). Chromatographic data were processed using the Empower™ Software System (Milford, MA, USA).

PM and PBAlc were analyzed using the reversed phase HPLC method modified from the previous study [25]. The Fortis™ C_18_ column (150 × 4.6 mm i.d., 5 µm particle size) connected to a Sunfire™ C_18_ guard column (20×4.6 mm i.d., 5µm particle size) was used for separation. The freshly prepared mobile phase was water and acetonitrile. Elution was performed using the gradient program starting at 65% acetonitrile for 5 min, then increased to 100% acetonitrile from 5 to 15 min, and decreased to 65% acetonitrile from 15 to 20 min, with a post run of 7 min to equilibrate the column between injections. The flow rate of the eluent was constant at 1.4 mL/min. The column temperature was maintained at 25±2°C. The injection volume was 20 μL. The analytes were detected at a wavelength of 230 nm.

### 2.6. Method Validation

The method of analysis was validated in accordance with the USFDA Guidance [26] in terms of linearity, precision, accuracy, recovery, and lower limit of quantification (LLOQ).

Linearity was determined by preparing standard plasma samples with different concentrations of PM (0.1, 0.2, 0.4, 0.8, 1.6, and 3.2 µg/mL; *n*=4) and PBAlc (0.1, 0.8, 6.4, 51.2, 102.4, and 204.8 µg/mL; *n*=4). The calibration curves were constructed by plotting the peak area of the analyte (*y*) against its concentrations (*x*). Regression analysis for each calibration curve was performed to obtain the calibration equation and correlation coefficient (*r*).

Precision was evaluated by using four quality control (QC) samples of PM (0.1, 0.2, 0.8, and 3.2 µg/mL) and PBAlc (0.1, 0.8, 51.2, and 204.8 µg/mL) that were prepared by adding different concentrations of standard solutions into the plasma blank samples. Intra-day precision was determined by assaying five samples of each concentration during the same day while the inter-day precision was determined by assaying the samples daily for five consecutive days. The precision was expressed as the relative standard deviation (RSD) and was calculated using the formula: $RSD\;(\%)=\frac{\text{standard deviation}}{mean}\times 100$. The level of acceptance was within 15%RSD except at the LLOQ where 20%RSD is acceptable.

The same concentration of QC samples was used for determining the accuracy. Intra-day accuracy was determined by analyzing five samples of each concentration on the same day while the inter-day accuracy was determined by assaying for five consecutive days. The accuracy was expressed as the deviation (DEV) and was calculated using the formula: $DEV(\%)=\frac{measured\;concentration-nominal\;concentration}{nominal\;concentration}\times 100$. It was acceptable when the DEV was within ±15% or ±20% at the LLOQ.

Recovery by the extraction was determined at concentrations of 0.1, 0.2, 0.8, and 3.2 µg/mL for PM and 0.1, 0.8, 51.2, and 204.8 µg/mL for PBAlc (*n*=5). The recovery was calculated using the expression: $Recovery(\%)=\frac{response\;after\;extraction}{response\;after\;direct\;injection}\times 100$.

The LLOQs of PM and PBAlc were defined as the lowest concentration on the calibration curve that could be determined with a signal-to-noise ratio (*S*/*N*) of 5.

Validation results (Table 1) showed that calibration curves of PM and PBAlc were linear with good correlation coefficient (*r*≥0.9999). The method was shown to measure both trans-PM and PBAlc precisely and accurately. Both intra- and inter-day precisions were within the level of acceptance, *i.e.*,±15%RSD and ±20%RSD for the concentration at LLOQ [26]. PM and PBAlc were almost completely recovered from plasma, *i.e.*, ≥80% for *trans*-PM and ≥91% for PBAlc. The LLOQs for *trans*-PM and PBAlc were 0.1 µg/mL.

**Table 1:** Method validation for analysis of *trans*-permethrin (PM) and phenoxybenzylalcohol (PBAlc) in rat plasma.

Validation parameter		Analyte	
		*trans*-PM	PBAlc
Range (µg/mL)		0.1–3.2	0.1–208.4
Linearity	Slope	22491 ± 374.08	34378 ± 400.79
	*y*-Intercept	(−)239.08 ± 107.93	806.31 ± 419.26
	*r*	0.9999	1.0000
Precision (%RSD)	Intra-day	2.24–7.63	1.18–3.66
	Inter-day	3.12–6.75	2.02–3.46
Accuracy (%DEV)	Intra-day	(−)2.37–(+)10.92	(−)12.89–(+)1.79
	Inter-day	(−)7.85–(+)3.44	(−)11.91–(−)0.21
Recovery (%)		80.43–82.34	91.53–95.07
LLOQ (µg/mL)		0.1	0.1

### 2.7. Data Analysis

Semi-log plasma concentrations of *trans*-PM and PBAlc against time were plotted. The elimination rate constant (k_el_) was determined from the slope of the terminal phase (*T*_14_ − *T*_22_) of the curve following the equation: $k_{\text{el}}=\frac{-slope}{2.303}$. The elimination half-life (*t*_1/2 el_) was estimated according to the equation: $t_{1/2\text{el}}=\frac{0.693}{k_{\text{el}}}$.

The permethrin metabolic ratio (PMR) was calculated as the concentration ratio between PBAlc and *trans*-PM as follows: $PMR=\frac{\left[PBAlc\right]}{\left[trans-PM\right]}$. The change in PMR was calculated using the expression: $\%Change\;in\;PMR=\frac{\left(PMR_{phase\;II}-PMR_{phase\;I}\right)}{PMR_{phase\;I}}\times 100$.

All data were expressed as a mean±SEM. The phase I (without pretreatment) and phase II (after pretreatment) data were obtained from the same animal and were compared using paired *t*-test. Data derived from different groups of pretreatment were analyzed using one-way analysis of variance (ANOVA) followed by post hoc least significant difference test. A significant difference was considered to be *p* < 0.05. Statistical analyses were performed using SPSS Statistics Bass 17.0 for Windows EDU (SPSS Inc. Chicago, IL, USA).

## 3. Results

### 3.1. Determination of PM and PBAlc in Plasma

**Figure 3:**
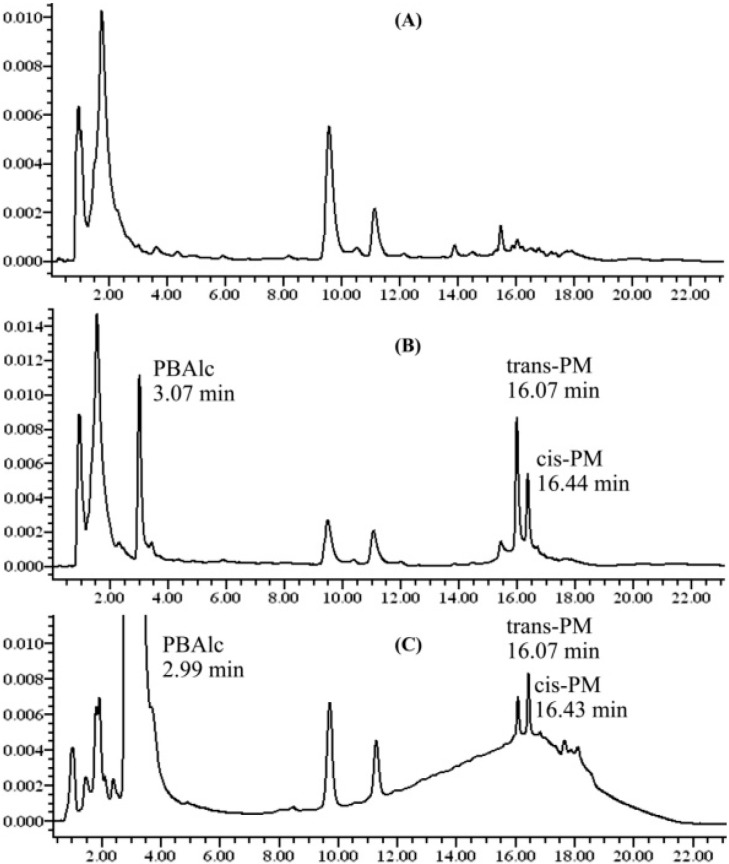
Representative chromatograms for the separation of PM and PBAlc in rat plasma; (**A**) blank plasma; (**B**) blank plasma spiked with standards of PM and PBAlc(3.2 µg/mL); (**C**) plasma sample at 8 h after dosing with PM (460 µg/mL, p.o.).

Chromatographic profiles (Figure 3) show that PBAlc, *trans*-PM, and *cis*-PM were well separated from plasma interferences with an elution time of approximately 17 min.

**Figure 4:**
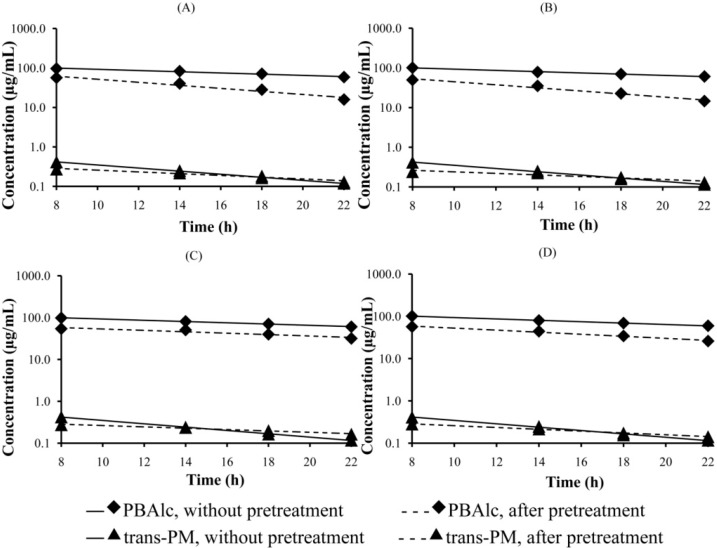
Semilog plasma concentration-time profiles of PBAlc and *trans*-PM during 8–22 h in rats receiving a single or multiple oral dose(s) of MG (40 mg/kg) and AE (100 mg/kg); (**A**) MG single dose; (**B**) MG multiple dose; (**C**) AE single dose; (**D**) AE multiple doses.

Plasma concentration-time profiles of *trans*-PM and PBAlc in rats pretreated with a single and multiple oral dose(s) of MG (40 mg/kg) and AE (100 mg/kg) are presented in Figure 4. PBAlc and *trans*-PM concentrations were measurable in the plasma during 8–22 h post dose and gradually declined over time. After 22 h, the levels of *trans*-PM in the plasma of rats given a single dose and multiple dose pretreatment of MG were below the LLOQ.

The plasma concentrations of *trans*-PM after pretreatment with a single and multiple dose(s) of MG were significantly decreased at 8 and 14 h post dose compared with those without pretreatment(Figure 4A,B). However, the *trans*-PM concentrations at 18 and 22 h were relatively increased. Similar findings were noted for rats pretreated with a single and multiple dose(s) of AE (Figure 4C,D). In contrast, the plasma concentrations of PBAlc after pretreatment with a single and multiple dose(s) of MG and AE were significantly decreased at 8, 14, 18, and 22 h.

The pharmacokinetic parameters that represent an elimination of *trans*-PM and PBAlc are presented in Table 2. Without pretreatment, the average values of the elimination rate constant (k_el_) of PM were similar and ranged from 0.059 to 0.062 h^−1^. The value of k_el_ was significantly decreased after pretreatment with a single dose of MG, *i.e.*, from 0.061 to 0.035 h^−1^. Likewise, the k_el_ for *trans*-PM was significantly reduced by administering multiple doses of MG and a single and multiple dose(s) of AE, *i.e.*, from 0.062 to 0.033 h^−1^, from 0.060 to 0.031 h^−1^, and from 0.059 to 0.024 h^−1^, respectively.The decrease in the k_el_ was the highest, *i.e.*, about 2.5-fold, in the group that received AE multiple dose pretreatment. In contrast to PM, the elimination rate constants of PBAlc were significantly increased after pretreatment with a single dose of MG (from 0.035 to 0.075 h^−1^), multiple doses of MG (from 0.035 to 0.107 h^−1^), and multiple doses of AE (from 0.035 to 0.059 h^−1^). The highest increase (3.1 folds) was observed after pretreatment with multiple doses of MG.

**Table 2:** Elimination rate constants (k_el_)^†^ and elimination half-lives (*t*_1/2 el_)^†^ of PM and PBAlc in rats following a single and multiple dose(s) of MG and AE administration.

Group	Substance	k_el_ (h^−1^)		*t*_1/2 el_ (h)	
		Without pretreatment	After pretreatment	Without pretreatment	After pretreatment
I (MG single dose)	PM	0.061 ± 0.001 ^a^	0.035 ± 0.002 ^*a^	11.46 ± 0.13	19.98 ± 1.17 ^*a^
	PBAlc	0.035 ± 0.004	0.075 ± 0.007 ^*A^	20.75 ± 2.13	9.49 ± 0.80 ^*A^
II (MG multiple dose)	PM	0.062 ± 0.001 ^a^	0.033 ± 0.002 ^*a^	11.19 ± 0.12	22.65 ± 0.85 ^*a^
	PBAlc	0.035 ± 0.002	0.107 ± 0.008 ^*B^	20.46 ± 0.82	6.65 ± 0.49 ^*A^
III (AE single dose)	PM	0.060 ± 0.001 ^a^	0.031 ± 0.001 ^*a^	11.61 ± 0.22	22.69 ± 0.68 ^*a^
	PBAlc	0.041 ± 0.003	0.042 ±0.005 ^C^	17.41 ± 1.34	17.80 ± 2.21 ^B^
IV(AE multiple dose)	PM	0.059 ± 0.001 ^a^	0.024 ± 0.001 ^*b^	11.71 ± 0.26	29.08 ± 1.47 ^*b^
	PBAlc	0.035 ± 0.001	0.059 ± 0.002 ^*AC^	20.06 ± 0.80	11.88 ± 0.48 ^*AC^

^†^ Mean ± SEM (*n*=6); *p < 0.01, compared with without pretreatment using paired *t*-test. Values within the same column that have different letter superscripts (lowercase for PM; uppercase for PBAlc) indicate a significant difference using ANOVA by post hoc least significant difference test, *p* < 0.01.

The baseline elimination half-lives of *trans*-PM in all groups of pretreatments ranged from 11.19 to 11.71 h (Table 2). Pretreatment with single and multiple doses of MG and AE significantly increased the elimination half-life of *trans*-PM, *i.e.*, from 11.46 to 19.98 h, 11.19 to 22.65 h, 11.61 to 22.69 h, and 11.71 to 29.08 h, respectively. The increase in *t*_1/2 el_ was the greatest (2.5-fold) following administration of multiple doses of AE. The elimination half-lives of PBAlc were significantly decreased after pretreatment with a single dose of MG (from 20.75 to 9.49 h), multiple doses of MG (from 20.46 to 6.65 h), and multiple doses of AE (from 20.06 to 11.88 h). The highest decrease (3.1 folds) was observed after pretreatment with multiple doses of MG.

**Figure 5:**
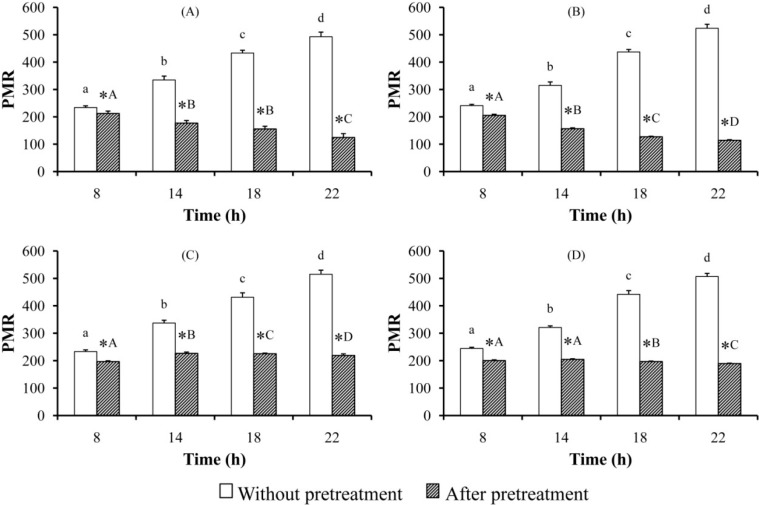
PMR^†^ in rats receiving a single and multiple oral dose(s) of MG (40 mg/kg) and AE (100 mg/kg); (**A**) MG single dose; (**B**) MG multiple doses; (**C**) AE single dose; (**D**) AE multiple doses. ^†^ Mean ± SEM. Significance differences were considered at *p* < 0.05. Asterisk represents significant difference when compared with without pretreatment at the same time using paired *t*-test. A different letter (a lowercase for without pretreatment; an uppercase for after pretreatment) indicates significant difference using ANOVA followed by least significant difference test.

Figure 5 represents the PMRs in rats without and after pretreatment with a single and multiple dose(s) of MG and AE. A single dose of MG caused a significant decrease in PMRs at 8, 14, 18, and 22 h after PM administration (Figure 5A). Similar results were observed in the pretreated rat groups receiving multiple doses of MG (Figure 5B), and single and multiple doses of AE (Figure 5C,D).

**Figure 6:**
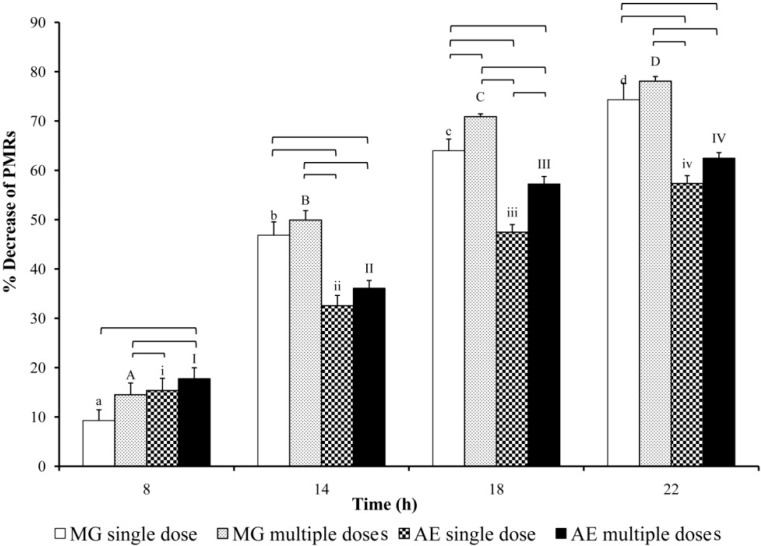
Percentage of the decrease in PMRs^†^ in rats following a single and multiple oral administration of MG (40 mg/kg) and AE (100 mg/kg). ^†^ Mean ± SEM. Significance differences were considered at *p* < 0.05 using ANOVA followed by least significant difference test. A different sign (a lowercase letter for MG single dose; an uppercase letter for MG multiple doses; a lower case roman number for AE single dose; an upper case roman number for AE multiple doses) indicates significant difference among the same treatment. The bracket indicates a pair of data having significant difference.

The percent decrease in PMRs is illustrated in Figure 6. The percentage of the decrease was significantly increased with time for all groups of pretreatment.

At 8 h, the percent reduction of PMRs tended to increase after pretreatment with multiple doses of MG or AE. It was the highest after the multiple-dose administration of AE. In contrast, at 14, 18, and 22 h, the percent reduction of PMRs was significantly decreased after a single- and multiple-dose administration of AE compared to those after single- and multiple-dose administration of MG.

The percent reduction in PMRs tended to increase after pretreatment with multiple doses of either MG or AE, compared with those after a single dose. The reduction was significant at 18 h after PM administration.

The results show that after pretreatment with MG or AE, the elimination of PM was slower, and the levels of the metabolite PBAlc was lower. These findings point to inhibited metabolism of PM.

## 4. Discussion

Hepatic metabolism of PM is responsible for its detoxification and elimination. The pathways and enzymes for PM metabolism have been documented in many *in vitro* studies. The study using human liver fractions showed that *trans*-PM was metabolized to PBAlc and phenoxybenzoic acid (PBAcid). To produce PBAlc, the ester bond of *trans*-PM was hydrolyzed by CES [27], a serine hydrolase catalyzing the hydrolysis of esters, amides, and thioesters [28]. PBAlc was then oxidized by alcohol dehydrogenase (ADH) to phenoxybenzaldehyde (PBAld) and subsequently to PBAcid by aldehyde dehydrogenase. *cis*-PM was not significantly metabolized. Both the hydrolysis of *trans*-PM and oxidation of PBAlc did not involve the cytochrome P450s. A study using rat microsomes showed that *trans*-PM was more effectively hydrolysed than *cis*-PM by liver microsomes and by the small intestinal microsomes of rats. The ES-3 and ES-10, isoforms of the CES1 family were responsible for the hydrolysis of *trans*-PM. Oxidation of the alcohol and aldehyde metabolites required cytochrome P450 [16]. Another study reported that metabolism of *trans*-PM in the rat and human liver microsomes were from the combination of both hydrolysis and oxidation while that of the *cis*-PM was solely the result of oxidation [29].

The MG and *M. speciosa* leaf extracts have been shown to interact with the drug-metabolizing enzymes. Based on human recombinant CYP enzymes, a methanolic extract of *M. speciosa* showed the most potent inhibition on CYP2D6 activity. The extract had a weak effect on CYP3A4 activity and a negligible effect on CYP2C9 activity [30]. It has been confirmed that MG had the strongest inhibitory activity on CYP2D6, while CYP2C9 and CYP3A4 are inhibited to a lesser extent [31]. An *M. speciosa* alkaloid extract had a moderate inhibitory effect on CYP1A2 [32], while MG exhibited an inductive effect on CYP1A2 [33]. In an *in vitro* study, the methanolic, aqueous, and total alkaloid extracts of *M. speciosa* leaves exhibited a concentration-dependent inhibition of glutathione *S*-transferase (GST), 61%, 50%, and 43% inhibition, respectively, in rats, whereas the *in vivo* findings showed an enzyme induction after a 14-day treatment [34]. MG inhibited GST in a dose-dependent manner, but a high dose was required to induce the activity of *N*-demethylase in both diabetic male and female rat liver cytosolic fractions [35]. A recent study confirmed that there was an increase in the activity of *N*-demethylase and UDP-glucuronosyltransferase (UGT) in male rats treated with the methanolic, aqueous, and total alkaloid extract from *M. speciosa* leaves [36].

There have been no reports that *M. speciosa* extract and MG alter CES activity, but this enzyme can be affected by several other chemicals. For example, dexamethasone and phenobarbital have been shown to induce CES in humans [37]. Organophosphate and carbamate insecticides have caused an enzyme inhibition through phosphorylation or carbamylation of the serine residue at the active center of the enzyme [15,38]. The only study that has identified metabolites of MG in rat and human urine postulated its metabolic pathways in both species [17]. MG was metabolized by hydrolysis of the methylesterat position 16 and *O*-demethylation of the 9-methoxy group and of the 17-methoxy group, followed by oxidation of the intermediate aldehydes to carboxylic acids or reduction to alcohols and a combination of some steps. No information about the enzymes responsible for the metabolism of MG has been reported. However, the substrate specificity of CES1 has been documented [39]. CES1 preferentially catalyses the hydrolysis of compounds esterified with a small alcohol group. That corresponds to the structure of MG and is consistent with hydrolysis of the methylester at position 16.It may be possible that MG binds to CES1 and is a substrate for hydrolysis. In addition, the results of the study suggest that MG causes inhibition of CES1, and thus inhibits the metabolism of PM. It would be possible and helpful to further investigate and confirm the interaction of MG with CES1, by determination of CES1 activity and possibly in appropriate *in vitro* studies.

The metabolic ratio in plasma and/or urine has been a valuable tool for enzyme activity evaluation [40]. Without pretreatment of MG and AE, PMRs increased with time because metabolite levels increased while PM levels decreased. After pretreatment, PMRs decreased and the extent of those decreases increased with time. This might be a result of the decrease of metabolite (PBAlc) levels and the increase of PM levels. As noted, there was a relative increase in *trans*-PM levels and a significant decrease in PBAlc levels during the terminal phase of PM elimination. The elimination kinetics shows that after pretreatment, PM was cleared more slowly while PBAlc was eliminated more rapidly. That is probably due to inhibition of PM hydrolysis catalyzed by CES and further metabolism of PBAlc to aldehyde and acid metabolites. Since the levels of PBAld and PBAcid were not measured, whether MG and AE induce activities of CYPs, ADH, and ALDH involving in conversion of PBAlc to PBAld and PBAcid is unclear.

Effects of single and multiple doses of either MG or AE administration on the PMR were similar. With repeated doses, the drug will accumulate in the body and reach a steady state until dosing stops. If the dosing interval is less than four half-lives, accumulation will occur. The accumulation is indexed by the accumulation factor, which equals one divided by the fraction lost in one dosing interval. When the drug is given once every half-life, the accumulation factor is 2. This means that the concentration after intermittent doses at steady state will be twice that at the same time after the first dose. A previous study [18] has reported an elimination half-life of 9.4 h after an oral dose of MG in rats. In this work, MG and AE were administered once a day, which is about 2.6 times its elimination half-life. The accumulation factor is 1.31. It is likely that the steady state levels after four doses were only slightly higher than after a single dose. Therefore, no major additional inhibitory effect can occur as compared to a single dose.

The decrease in PMR due to MG and AE in the present study was different as seen from the smaller extent of reduction in the PMR caused by AE compared with MG. With regard to the procedure for preparation of AE and MG [18,20,21], the yield of MG was reported to be 1.27 g per 2.5 g of AE, *i.e.*, 50.8%. The amount of MG in the AE preparation was 1.27 times higher, compared to that in the MG preparation. Hence, the different effects may be due to different active constituents in the preparations. MG was 98% pure [18], whereas AE contained the main indole alkaloid (MG) and other minor constituents such as speciogynine, speciociliatine, paynantheine, 7-hydroxymitragynine, including newly discovered compounds, like 7β-hydroxy-7*H*-mitraciliatine and isospeciofoleine [41]. Some of these minor substances may counteract the inhibitory effect of MG.

In the present study, we have described the elimination kinetic parameters in male Wistar rats following a single dose (460 mg/kg) of PM. The values of k_el_ and *t*_1/2 el_ without pretreatment for all groups were considered the baseline. The k_el_ (0.059−0.062 h^−1^) and *t*_1/2 el_ (11.19−11.71 h) obtained in this study were comparable to those from previous work [42]. The authors investigated the toxicokinetics of PM in male Sprague–Dawley rats and reported the rate constants for the terminal elimination phase (β) and the half-life at the β phase of 0.056 h^−1^ and 12.37 h, respectively, after an oral dose of 460 mg/kg of PM.

The toxic effects of PM appeared in the present study. The dose of PM (460 mg/kg) was below the acute oral LD_50_ (median lethal dose) value in the adult rats, *i.e.*, 1200–1500 mg/kg [14,43], and was not lethal to the animals. Following an oral administration of PM, the animals (without pretreatment) showed the typical symptoms for type-I pyrethroids toxicity such as hypersensitivity and tremors. These symptoms were exaggerated in the group that concurrently received either MG or AE and PM (data not shown). This may be due to the decrease in the rate of hydrolysis, which is important for the rate of degradation, and elimination of *trans*-PM. Plasma *cis*-PM was not determined in this work due to the lack of a pure standard. However, since hydrolysis of *cis*-PM is slower than that for the *trans*-isomer, and this isomer has a significantly higher affinity for the sodium channel than the *trans*-isomer, this may also contribute to the higher toxicity of PM [15].

## 5. Conclusions

MG and other alkaloids derived from *M. speciosa* exhibited toxicokinetic interactions with PM in rats. The interaction seems to be due to inhibition of PM hydrolysis presumably caused by CES, delaying PM elimination and enhancing PBAlc elimination. The findings suggest that humans consuming a kratom cocktail might be at higher risk of developing neurotoxicity from PM.
